# Principles of the Theory and Practice of Medicine

**Published:** 1839-10-26

**Authors:** 


### BIBLIOGRAPHICAL NOTICE.

*Principles of the Theory and Practice of Medicine.* By MARSHALL HALL, M. D., F. R. S., Lecturer on the Theory and Practice of Medicine at the Webb Street School of Medicine, &c. First American Edition. Revised and much enlarged, by JACOB BIGELOW, M. D., Professor of Materia Medica in Harvard University, &c.; and OLIVER WENDELL HOLMES, M. D., Member of the Medical Society of Observation of Paris, &c. Boston: 1839. 8vo., pp. 724.

THIS work, our readers will perceive, is ushered into the world under the auspices of Professors Bigelow and Holmes. From our knowledge of the critical judgment and professional acquirements of these gentlemen, we know that a book which they thus recommend to the notice of the profession, must be good. The only question would be as to its relative excellence, compared with other works of a similar kind.

Dr. Hall has evidently founded his work on *practice*, in a great degree, upon the previous essay on diagnosis, which is almost included in it. The same arrangement of the matter into short, condensed paragraphs, is followed, with frequent tabular views of the symptoms and lesions. This method possesses the great advantages of being well fitted for reference, condensed, and very clear; it has the disadvantages of being dry and fatiguing to the mere reader. It will, then, at once be perceived, that the object of the work is to furnish a valuable standard of reference to the student, to which he may always refer—certain of meeting with information which is nearly, if not absolutely, in conformity with the actual state of our knowledge on the subject. Hence, to the student it is invaluable; for it is free from theory, clear, easily consulted, and a definite impression is left upon the mind of the reader, which is far from being the case with all general works upon practice. Condensed as the volume appears, it is the most complete treatise within the reach of most readers.

The breaking up of the chapters into short paragraphs, in which the style is reduced to the least possible number of words capable of expressing an idea, will not make the work of Dr. Hall a favourite with the pupil who is unwilling to bend his mind to the serious contemplation of the profession into which he is thoughtlessly entering; but we believe that the number of those who are apparently incapable of the mental effort required to grapple with a dry, but elaborate work, is not great enough to interfere materially with its circulation. On the contrary, we can promise for it a large demand amongst the pupils and practitioners of the middle states.

The present state of the science is reached in nearly every chapter. The copious notes of the American editors have supplied some deficiencies. In the chapter on the exanthemata, there is still some deficiency. The description of the lesions of the internal organs in these fevers, is short and unsatisfactory. The chapters on tuberculous disease are also rather less perfect than other parts of the work. In that relating to the inflammation of the membranes of the brain in children, there occurs an omission which would have been supplied by the editors but for an unforeseen accident.

Although these chapters are a little less perfect than could have been desired, and some other portions of the work are objectionable, the excellence of the whole is by no means diminished, and we would unhesitatingly pronounce it the best and most complete text-book for the study of the practice of medicine. It is full of facts, well arranged and digested, and free from the endless repetitions, and diffuse, ill digested matter, which are often introduced into treatises upon the practice of medicine. In them, more than in any other scientific works, we require the greatest order and precision of arrangement, and the most complete separation of the doubtful from the known and admitted truths.

